# The effects of glyphosate, glufosinate, paraquat and paraquat-diquat on soil microbial activity and bacterial, archaeal and nematode diversity

**DOI:** 10.1038/s41598-018-20589-6

**Published:** 2018-02-01

**Authors:** Paul G. Dennis, Tegan Kukulies, Christian Forstner, Thomas G. Orton, Anthony B. Pattison

**Affiliations:** 1 School of Earth and Environmental Sciences, The University of Queensland, Brisbane, QLD 4072 Australia; 2 Department of Agriculture and Fisheries, Centre for Wet Tropics Agriculture, 24 Experimental Station Road, South Johnstone, QLD 4859 Australia

## Abstract

In this study, we investigated the effects of one-off applications of glyphosate, glufosinate, paraquat, and paraquat-diquat on soil microbial diversity and function. All herbicides were added to soil as pure compounds at recommended dose and were incubated under laboratory conditions for 60 days. High-throughput phylogenetic marker gene sequencing revealed that none of the herbicides significantly influenced the richness, evenness and composition of bacterial and archaeal communities. Likewise, the diversity, composition and size of nematode communities were not significantly influenced by any of the herbicides. From a functional perspective, herbicides did not significantly affect fluorescein diacetate hydrolysis (FDA) and beta-glucosidase activities. Furthermore, the ability of soil organisms to utilise 15 substrates was generally unaffected by herbicide application. The only exception to this was a temporary impairment in the ability of soil organisms to utilise three organic acids and an amino acid. Given the global and frequent use of these herbicides, it is important that future studies evaluate their potential impacts on microbial communities in a wider-range of soils and environmental conditions.

## Introduction

Modern agriculture is dependent on the use of herbicides to control weeds that are a persistent threat to crop productivity. These chemicals can harm non-target organisms^1,2^, which may reduce biodiversity and the provision of ecosystem services that support food security and on-farm profitability. Soil microorganisms play central roles in the degradation of herbicides and drive terrestrial ecosystem functioning^1^. At present, however, little is known concerning the extent to which herbicides influence the diversity and function of these communities. This problem is particularly acute when considering the effects of the active constituents of commercially available herbicides, which are currently the only components that are typically disclosed.

Along the tropical north east coast of Australia, glyphosate, glufosinate, paraquat, and paraquat combined with diquat are among the most widely used herbicides in banana production. Glyphosate is the world’s most commonly used herbicide, and its effects on soil microbial communities when applied as various formulations of Roundup have been more extensively studied than any other herbicide.

Roundup has been shown to negatively affect fungal growth *in-vitro*^3^ and reduce root colonisation by beneficial bacteria^4^. Most studies, however, indicate that, when applied at or below the recommended field-rate (50 mg/kg), Roundup exerts negligible^5–10^ or minor^11–13^ effects on microbial community structure, and negligible effects on functionality as demonstrated by community-level substrate utilisation profiling^5^. Similarly, glyphosate applied as a pure compound, has been shown to have negligible effects on microbial community structure and function^14^.

For glufosinate, which is a chemical relative of glyphosate, some studies indicate that low-levels (≤10 mg/kg) exert significant positive and negative effects on the relative abundances of various bacterial taxa when applied as Basta^15,16^ and Liberty^17^, while others demonstrate that similar application rates of Basta^18^ and Liberty^19^ have no effect on microbial community structure.

When applied as Gramoxone or as a pure compound, paraquat has been shown to suppress culturable soil bacteria^20^ (Gramoxone), reduce dehydrogenase activity^20,21^ (Gramoxone), increase urease activity^22^ (pure) and exert both positive^22^ (pure) and negative^20^ (Gramoxone) effects on soil fungi and invertase activity^22^ (pure). Paraquat is also mixed with diquat in commercial herbicides, which have been observed to increase soil fungi^23^ (Sprayseed), reduce soil nematodes and bacteria, and suppress nematode diversity^24^ (Preeglone Extra).

Most studies concerning the effects of herbicides on soil microbial diversity are based on culturing, microbial fatty acid analyses (e.g. phospholipid fatty acid (PLFA) analysis) or fingerprinting (e.g. denaturing gradient gel electrophoresis (DGGE) and terminal-restriction fragment length polymorphism (T-RFLP) analyses) of phylogenetic marker genes. These methods can be used to compare the structure of microbial communities, but are not appropriate for estimation of alpha diversity (i.e. the numbers of taxa present and the evenness of their abundances)^25,26^. For culture-dependent analyses, this problem is related to the fact that only a minority of bacterial and archaeal taxa can be grown using standard cultivation methods^27,28^. For fatty acid^25^ and DNA fingerprinting-based analyses^26^, the problem is that multiple taxa may contribute to individual peaks or bands. Sequencing of phylogenetic marker genes circumvents most of these issues, facilitates estimation of alpha diversity and allows identification of the taxa present. Albeit infrequently, this approach has been used to characterise soil microbial responses to glyphosate as Roundup^10,12,29^ and glufosinate as Basta^30^. These studies indicate that neither herbicide influences the alpha diversity of soil bacterial communities. To our knowledge, phylogenetic marker gene sequencing has never been applied to characterise soil microbial responses to glyphosate and glufosinate as pure compounds or to paraquat and paraquat-diquat as pure compounds or commercial formulations.

Another caveat of most studies concerning the effects of herbicides on soil microbial diversity is that they tend to focus on one trophic level. Nematodes may exist at different trophic levels and are indicative, therefore, of food web complexity^31^ especially when combined with analyses of other groups such as bacteria and archaea. The generation time of nematodes is longer than soil bacteria, making them more stable to soil environmental changes^32^. Soil nematodes have been used extensively as bioindicators of soil functioning^32,33^ and environmental disturbances, including soil chemical applications^34–36^. In a meta-analysis of 18 studies focussing on soil nematode responses to different herbicides, Zhao *et al*.^37^ found that the total numbers of nematodes as well as the frequencies of bacterivorous, plant parasitic and omnivorous nematodes increased, while those of fungivorous and predatory nematodes decreased. Nonetheless, of the 18 studies included only one focussed on glyphosate^38^, one considered glufosinate^39^ and two investigated paraquat^40,41^. Liphadzi *et al*.^38^ found that nematode densities and trophic group responses did not differ between control and glyphosate treated soils. For glufosinate, which was applied to soil as Basta, Griffiths *et al*.^39^ observed small but variable effects on total nematode abundance; however, these effects were reported to be small compared with other standard management practices and were only detected in one of two soils tested. Lastly, Ishibashi *et al*.^40^ did not observe any significant effects of paraquat addition to soil on the numbers of total, plant parasitic, free-living or predatory nematodes.

In this study, we investigated the effects of one-off applications of glyphosate, glufosinate, paraquat, and paraquat-diquat at recommended doses (Table 1), on the diversity and function of bacterial, archaeal and nematode communities associated with a soil collected from a banana plantation in the wet tropics of north east Queensland, Australia. Soils were incubated in containers for 60 days and communities were characterised at multiple time points. The diversity of bacterial and archaeal communities was characterised by Illumina MiSeq sequencing of 16 S rRNA gene amplicons, while that of nematode communities was determined using microscopy. The functioning of microbial communities was characterised by measuring fluorescein diacetate hydrolysis (FDA) and beta-glucosidase activities, as well as the induced respiratory responses to 15 substrates^42^. These parameters are strongly associated with biogeochemical cycling and respond rapidly to soil environmental change^43^. FDA hydrolysis, for example, is a measure of total microbial enzyme activity^44^, while beta-glucosidases catalyse the hydrolysis of beta-glucosides (e.g. cellobiose), which represents the final, and often rate-liming step, in the degradation of cellulose – the world’s most abundant plant polymer^43,45^. Lastly, the induced respiratory responses of soil microbial communities to different substrates reflects their catabolic capabilities and can be measured using MicroResp^42^, which facilitates measurement of CO_2_ efflux directly from soil in multi-well plates. This approach circumvents the need to extract and culture soil organisms, which are key disadvantages associated with other methods for community level physiological profiling (CLPP)^46^. We used these data to test the hypothesis that one-off applications of the herbicides, as pure compounds and at recommended dose, influence the diversity and function of soil bacterial, archaeal and nematode communities.

**Table 1:** Application rates for each herbicide active.

Herbicide treatment	Herbicide active	Corresponding commercial herbicide	Active concentration in commercial herbicide (g/L or kg)	Upper limit of recommended application rate for each commercial herbicide (L or kg/ha)	Concentration of herbicide active applied to soil (ppm)
1) Glyphosate	Glyphosate	Roundup	360	9.00	33.03
2) Glufosinate	Glufosinate	Basta	200	5.00	10.19
3) Paraquat	Paraquat	Gramoxone	250	3.20	8.16
4) Paraquat-diquat	Paraquat	Sprayseed	135	3.20	4.40
4) Paraquat-diquat	Diquat	Sprayseed	115	3.20	3.75

## Results and Discussion

### Bacterial and archaeal diversity

**Figure 1:**
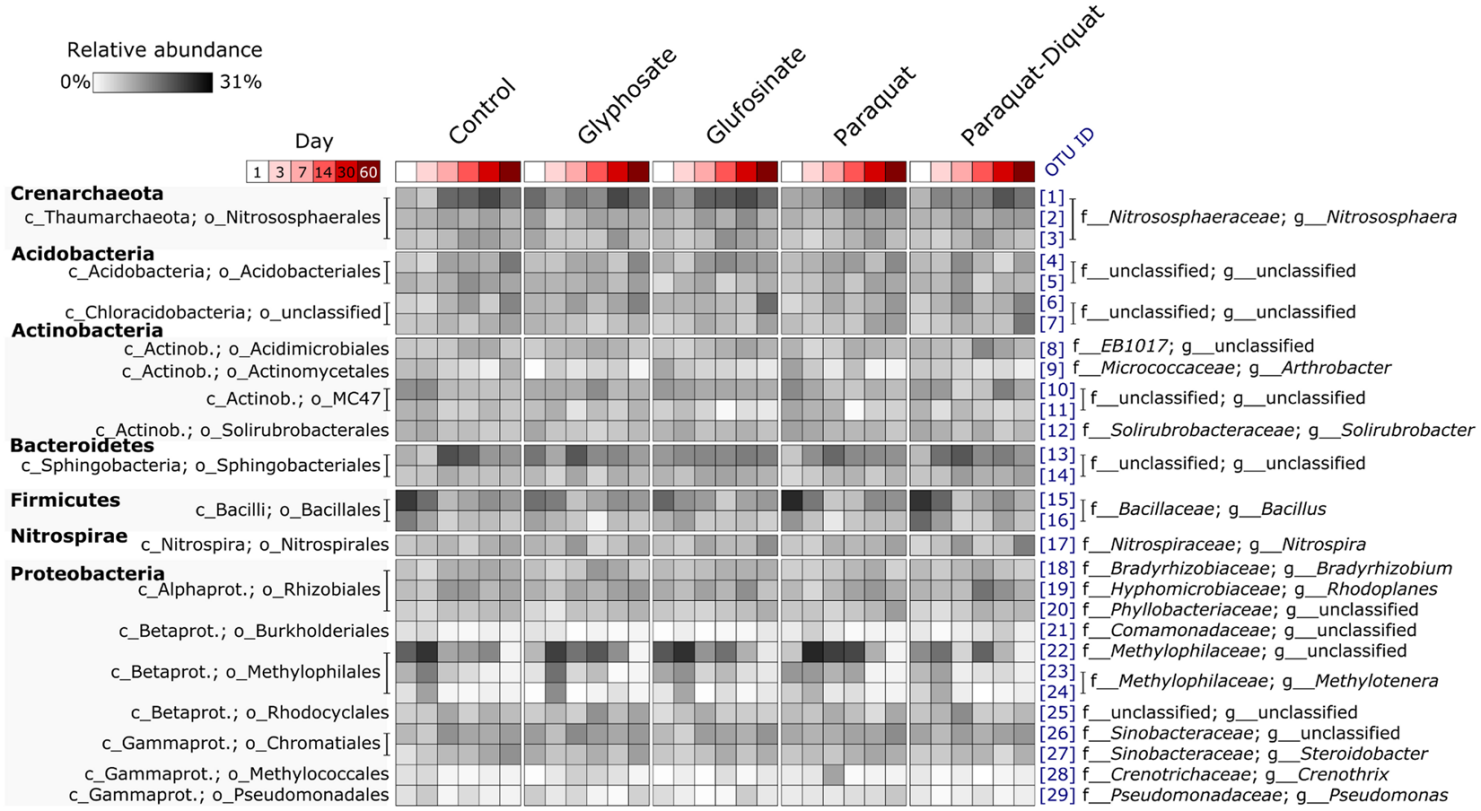
Heatmap summarising the composition of soil bacterial and archaeal communities overtime within treatments. The OTUs listed are those present at ≥1% average relative abundance in any treatment. The cell values in each column represent the mean averages of four replicate samples.

Soil bacterial communities were dominated by members of the Proteobacteria, Firmicutes and Bacteroidetes, while archaeal communities were dominated by representatives of the Chrenarchaeota (Fig. 1). Members of the Acidobacteria, Actinobacteria and Nitrospirae also constituted a significant fraction of the community in each soil (Fig. 1).

**Figure 2:**
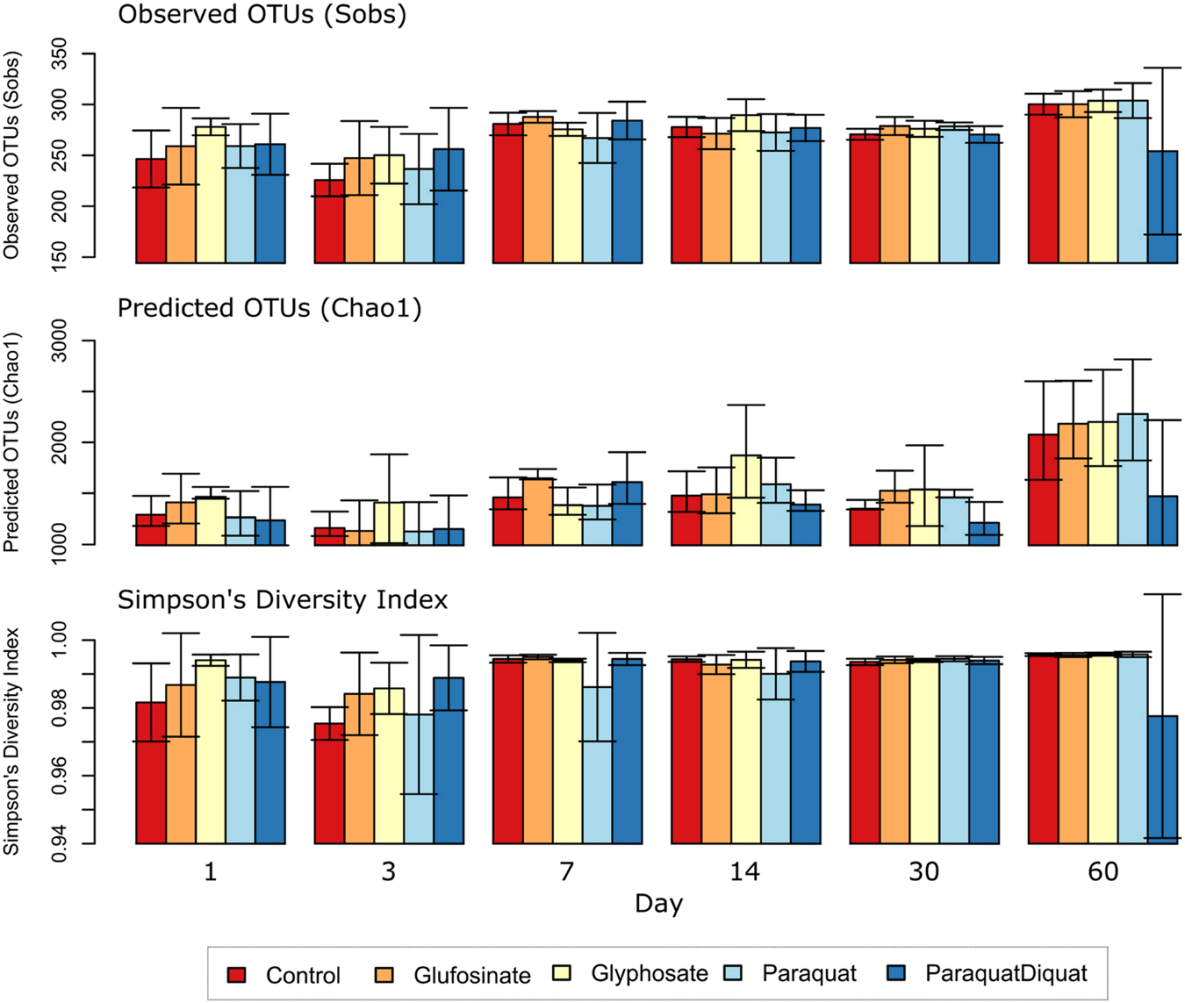
The alpha diversity of soil bacterial and archaeal communities over time. Each bar represents the mean average (n = 4) and the error bars represent standard deviations.

Relative to the controls, none of the herbicides led to significant changes in the richness (observed OTUs and Chao1 richness estimates) or evenness (Simpson’s Diversity Index) throughout the course of the experiment (Fig. 2). These findings are consistent with previous phylogenetic marker gene sequencing studies that found negligible effects on the alpha diversity of rhizosphere bacterial communities in response to: 1) glyphosate, applied at recommended dose in the form of Roundup PowerMax^29^ or Roundup Plus^10,12^, and 2) glufosinate, applied at recommended dose in the form of Basta^30^. We are not aware of any studies that used phylogenetic marker gene sequencing to characterise the alpha diversity of soil microbial communities in response to paraquat and paraquat-diquat, or their commercial formulations. In addition, our study is the first, to our knowledge, to investigate the effects of glyphosate, glufosinate, paraquat, and paraquat-diquat on the alpha diversity of soil microbial communities as pure compounds.

Our results indicate that, relative to the controls, the composition of bacterial and archaeal communities was not significantly affected by any of the herbicides. Similarly, Newman *et al*.^29^ did not observe significant changes in the relative abundances of Proteobacteria (*P* = 0.096) and Acidobacteria (*P* = 0.445) in response to glyphosate applied as Roundup PowerMax at recommended dose. Likewise, using 16S rDNA pyrosequencing, Barriuso *et al*.^10^ did not detect a significant change in community composition in response to glyphosate applied at recommended dose in the form of Roundup Plus. A range of other studies, using microbial fatty acid analysis (e.g. PLFA) or DNA fingerprinting (e.g. DGGE and T-RFLP), demonstrate that when applied at or below the recommended field-rate, the effects of pure glyphosate on microbial community structure are negligible^14^, while those of Roundup range from negligible^5–10^ to minor^11–13^.

Information concerning the effects of glufosinate on microbial community composition is limited. As seen in our study, recommended doses of glufosinate in the form of Basta and Liberty have been shown to have negligible effects on microbial community composition as represented by PLFA^18^ and DNA fingerprinting^19^, respectively. For paraquat and paraquat-diquat, information concerning their effects on soil microbial community composition is even more limited. In fact, we are not aware of any studies that investigated their effects on microbial community composition using culture independent methods.

### Nematode community structure

**Figure 3:**
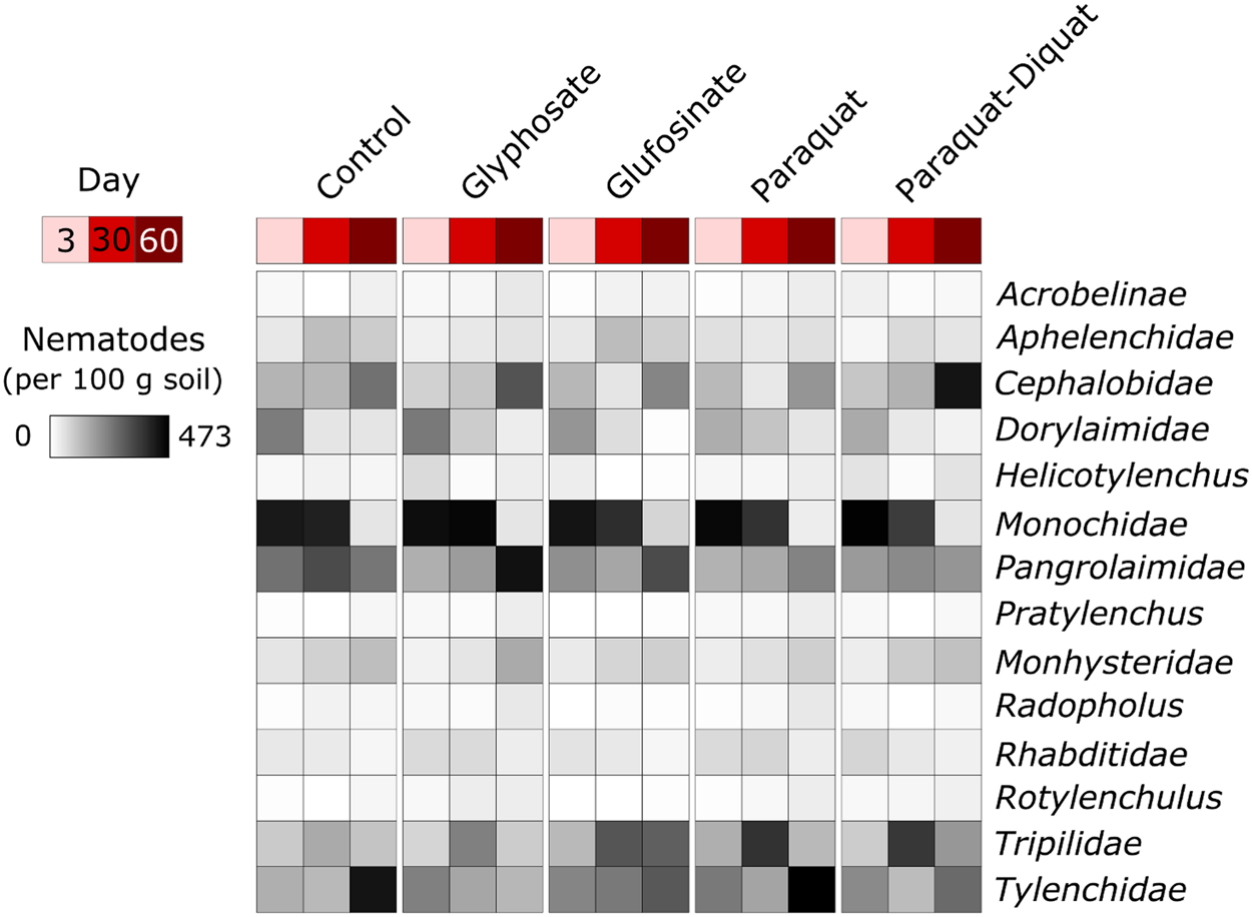
Heatmap summarising the abundances of nematode groups in each treatment. Each cell represents the average number of nematodes per 100 g of soil (n = 4).

Nematode communities were dominated by representatives of the families: *Monochidae*, *Pangrolaimidae*, *Tripilidae*, *Tylenchidae*, *Cephalobidae* and *Dorylaimidae* (Fig. 3). Relative to the controls, none of the herbicides led to a significant change in the total numbers of nematodes, or to the diversity (Simpson’s Diversity Index) and composition of nematode communities. Similarly, relative to the control, none of the herbicides led to significant changes in nematode trophic groups or guilds. Our findings are consistent with previous studies that found negligible effects of glyphosate^38^, glufosinate as Basta^39^, paraquat^40^, and paraquat-diquat as Sprayseed 250^47^ on nematode community composition, at recommended application rates.

### Microbial activity

**Figure 4:**
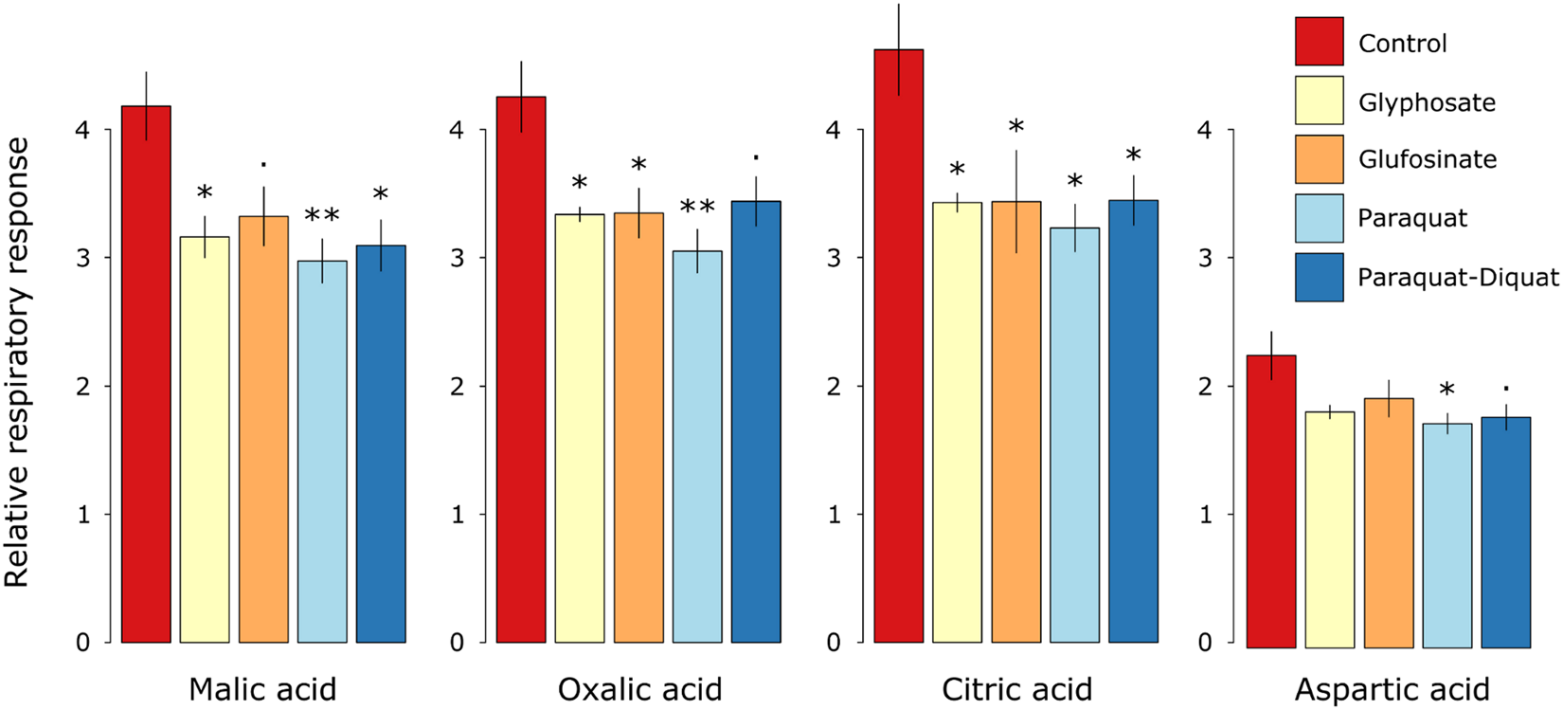
Inhibitory effects of herbicides on the respiratory responses of soil microbial communities to added substrates after seven days exposure. Asterisks indicate significant differences between control and herbicide treated soils (*P* < 0.10^**∙**^, *P* < 0.05*, *P* < 0.05**, Tukey’s HSD). Error bars represent standard errors of the means (n = 4).

From a functional perspective, our results indicate that: 1) none of the herbicides influenced total microbial enzyme activity (FDA) or beta-glucosidase activity; and that 2) while herbicide addition temporarily impaired the ability of soil organisms to utilise three organic acids and an amino acid (Fig. 4), their effects were otherwise negligible.

Our findings are in agreement with previous studies that found no changes in FDA^11^ and beta-glucosidase^48^ activity, as well as substrate utilisation patterns^5,14^ in response to recommended application rates of glyphosate. In contrast, Araújo *et al*.^49^ and Panettieri *et al*.^50^ found that recommended rates of glyphosate and GLYFOS Ultra (36% glyphosate) stimulated FDA and beta-glucosidase activity, respectively. This suggests that the effects of glyphosate on soil microbial activity can differ between soils.

For glufosinate, our findings are consistent with those of Zablotowicz *et al*.^51^, who observed no effects of one, two and four-times the recommended dose of glufosinate (i.e. 10, 20 and 40 ppm) on soil FDA activity, after two, four and seven days exposure. After one day, however, they observed a slight reduction in FDA activity at recommended dose and larger reductions at higher doses^51^. We are not aware of other studies that have characterised FDA and beta-glucosidase activities in response to paraquat and paraquat-diquat application. Likewise, we are not aware of previous studies that have investigated the effects of glufosinate, paraquat, and paraquat-diquat on the induced respiratory responses of soil microbial communities to multiple substrates.

The herbicide treatments significantly influenced community level physiological profiles on Day 7 (*P* = 0.03). This shift in response to herbicide addition was related to a reduction in the ability of soil organisms to utilise at least two organic acids (Fig. 4). Organic acids released by roots have been shown to play an important role in the ability of plant growth-promoting rhizobacteria to colonise banana roots^52^. Hence, it is possible that glyphosate, glufosinate, paraquat, and paraquat-diquat may reduce colonisation of roots by beneficial soil organisms. Nonetheless, our results indicate that organic acid utilisation was impaired only temporarily, hence limiting the likelihood of potential alterations in rhizosphere recruitment.

### Conclusions

In response to recommended application rates of glyphosate, glufosinate, paraquat, and paraquat-diquat as pure compounds we observed no significant effects on: 1) the richness, evenness and composition of soil bacterial and archaeal communities; 2) the composition of nematode communities; 3) soil FDA and beta-glucosidase activities; and 4) the induced respiratory responses of soil microbial communities to multiple substrates, except for three organic acids and an amino acid at Day 7 only. Within the context of our short-term, laboratory-based experiment, these findings indicate that single applications of these herbicide actives at recommended dose pose little threat to soil biodiversity and function. In practice, however, herbicides are applied multiple times per year on farms with different soils and environmental conditions, and commercial formulations contain a range of additional compounds that are not disclosed. Indeed the soil used in this study was collected from a banana plantation, where the herbicides tested in this study are in regular use. It is possible, therefore, that herbicide-intolerant organisms were already depleted. For this reason, despite being largely consistent with previous studies, our results must be interpreted with caution. Further work is essential to more fully understand the potential impacts of herbicides on soil microbial communities.

## Materials and Methods

### Experimental design and soil sampling

Soil was collected using a 5 cm diameter corer to a depth of 10 cm from a banana (*Musa acuminata* AAA var. Williams) paddock in East Palmerston, Queensland, Australia (S17° 35′32″ E 145° 49′ 58″), then transported to the Centre for Wet Tropics Agriculture, South Johnstone, Australia for further processing. The site is typical of the hydrological catchments that drain to the Great Barrier Reef lagoon. The soil had a clay loam texture (38% sand, 30% silt, 33% clay) and a pH in water of 6.7. The soil was passed through a 4 mm stainless steel sieve, adjusted to 50% water holding capacity and then 1.6 kg of soil was loaded into each of 44, 2 L plastic containers with lids that allowed gas exchange. These minicosms were incubated at 27 °C for 14 days prior to the application of treatments to allow the soil to equilibrate following physical disturbance. To maintain soils at 50% water holding capacity, the containers were weighed on a weekly basis, to calculate evaporative losses, which were corrected for by addition of water.

A total of five treatments were applied (four herbicides and a control). Each treatment was replicated four times and was arranged in a randomised block design. Our treatments included glyphosate, glufosinate, paraquat and an equimolar concentration of paraquat:diquat, which were solubilised in 10 ml water. For the control we included a 10 ml water-only treatment. Each herbicide active was applied at a dose corresponding to the upper recommended rates for the commercial herbicides that contain them (Table 1). To achieve this, the quantity of active compound that would be applied per cm^2^ of soil at the upper recommended rate was first multiplied by the exposed surface area of soil in each container (408 cm^2^). This number was then multiplied by four, as the depth of soil in each container was 4 cm and the bulk density of the soil was c. 1 g/cm^3^. All treatments were applied to the surface of the soil using a fine mist sprayer. Soils were incubated at 27 °C for 60 days.

For Microresp and characterisation of bacterial and archaeal diversity sub-samples of soil were collected from each container after 1, 3, 7, 14, 30 and 60 days incubation as soil cores using sterile 50 ml plastic tubes. Samples for DNA extraction were transferred to −20 °C storage and those for Microresp were maintained at 4 °C for 2–4 days until further processing. For nematodes, 200 g of fresh soil was collected from each container after 3, 30 and 60 days and extraction was performed immediately. Samples for enzyme assays were collected after 3, 7, 14 and 30 days and were maintained at 4 °C for 2–4 days until further processing.

### Characterisation of bacterial and archaeal communities

#### DNA extraction, PCR and sequencing

DNA was extracted from 250 mg (fresh weight) of thawed soil using the Power Soil DNA Isolation kit (MO BIO Laboratories, Carlsbad, CA) according to the manufacturer’s instructions. Universal bacterial and archaeal 16 S rRNA genes were amplified by polymerase chain reaction (PCR) using the primers 926F^53^ (5′-AAA CTY AAA KGA ATT GRC GG-3′) and 1392wR^53^ (5′-ACG GGC GGT GWG TRC-3′), each modified on the 5′ end to contain the Illumina overhang adapter for compatibility with the P5 and i7 Nextera XT indices, respectively. PCRs were performed on 1.5 µl DNA samples, in 1× PCR Buffer minus Mg^2+^ (Invitrogen), 100 µM of each of the dNTPs (Invitrogen), 300 µM of MgCl_2_ (Invitrogen), 0.625 U Taq DNA Polymerase (Invitrogen), 250 µM of each primer, made up to a total volume of 25 µl with molecular biology grade water. Thermocycling conditions were as follows: 94 °C for 3 min; then 35 cycles of 94 °C for 45 sec, 55 °C for 30 sec, 72 °C for 1 min 30 sec; followed by 72 °C for 10 min. Amplifications were performed using a Veriti^®^ 96-well Thermocycler (Applied Biosystems).

Amplicons were purified using Agencourt AMPure magnetic beads and subjected to dual indexing using the Nextera XT Index Kit (Illumina) according to the manufacturer’s instructions. Indexed amplicons were purified using Agencourt AMPure XP beads and then quantified using a PicoGreen dsDNA Quantification Kit (Invitrogen). Equal concentrations of each sample were pooled and sequenced on an Illumina MiSeq at The University of Queensland’s Institute for Molecular Biosciences (UQ, IMB) using 30% PhiX Control v3 (Illumina) and a MiSeq Reagent Kit v3 (600 cycle; Illumina) according the manufacturer’s instructions.

#### Processing of sequence data

Primer sequences were removed from each fastq file using the QIIME^54^ v1.9.1 script multiple_extract_barcodes.py. The header line of each sequence was then modified to contain a sample ID using a custom bash script and each file was quality filtered using the QIIME script multiple_split_libraries.py with the homopolymer filter deactivated. The forward reads from each sample were then concatenated into a single file and checked for chimeras against the October 2013 release of the GreenGenes database^55^ using UCHIME ver. 3.0.617^56^. Sequences were clustered at 97% similarity using UCLUST v. 1.2.22^57^. GreenGenes (October 2013 release) taxonomy was then assigned to the representative OTU sequences using BLAST+ v. 2.2.30. Data were rarefied to 350 sequences per sample for all comparisons of diversity. The mean numbers of observed OTUs, the estimated total OTUs (Chao 1) as well as Simpson’s Diversity Index values were calculated using QIIME.

### Characterisation of nematode communities

Nematodes were extracted using a modified Baermann funnel technique^58^, in which mesh baskets, each containing a single sheet of tissue paper and 200 g fresh soil, were placed in metal trays with 250 ml of deionised water. After 48 hours the soil was discarded and the solution was passed through a 25 µm sieve and backwashed twice with 10 ml of deionised water to collect the nematodes. From each 20 ml soil extract a 1 ml sub-sample was used to determine the total nematode abundance at low magnification, and then at higher magnification, 100 nematodes were identified to family-level^59,60^. Nematode abundances were expressed as numbers per 100 g of fresh soil. Nematodes were also categorized into feeding groups (i.e. fungivores, bacterivores, plant parasites, predators and ominvores) and functional guilds, which delineate feeding groups on the colonizer-persister (cp) scale according to their r and K characteristics (where cp = 1–5 and larger numbers represent more K dominated taxa)^35,61^. The groupings were as follows: Tylenchidae (fungivore, Fu2), Aphelenchidae (fungivore, Fu2), Rhabditidae (bacterivore, Ba1), Pangrolaimidae (bacterivore, Ba1), Cephalobidae (bacterivore, Ba2), Acrobelinae (bacterivore), Monhysteridae (bacterivore, Ba3), Tripilidae (carnivore, Ca3), Monochidae (carnivore, Ca4), Dorylaimidae (Omnivore, Om4), Helicotylenchus sp. (plant parasites, Pp3), Rotylenchulus sp. (plant parasites, Pp3), Radopholus sp. (plant parasites, Pp4), Pratylenchus (plant parasites, Pp4).

### Enzyme assays

Fluorescein diacetate (FDA) hydrolysis assays were used to provide a measure of total microbial enzyme activity and were performed using a modified version of the method initially proposed by Schnürer and Rosswall^44^. Briefly, 2 ml deionized water was added to 5 g fresh soil in 50 mL centrifuge tubes and incubated at 27 °C for seven days. Post-incubation, soils were shaken for 30 min in 20 ml 60 mM potassium phosphate buffer and 200 μl fluorescein diacetate solution (2000 μg/ml). Reactions were terminated by addition of 20 ml acetone.

Beta-glucosidase assays were performed as a measure of organic matter degradation potential using a modified method of Eivazi and Tabatabai^45^, in which the modified universal buffer and toluene were replaced with McIlvaine buffer (pH 6, 0.2 M dibasic sodium phosphate solution and 0.1 M citric acid) and 0.1% Tween buffer, respectively.

### Community-level physiology profiling

Community-level physiology profiles (CLPPs) were generated by characterising the induced respiratory responses of organisms associated with 400 mg (fresh weight) of each soil sample to 15 substrates using MicroResp^42^. The substrates included carboxylic acids (malic, oxalic, citric and fumaric acid), amino acids (L-alanine, DL-aspartic acid, γ-aminobutyric acid, L-lysine hydrochloride, L-arginine), carbohydrates (L-arabinose, D-fructose, D-galactose, D-glucose) and a phenolic compound (protocatechuic acid ethyl ester). Sterile distilled water was added to controls.

### Statistical analyses

Univariate responses were analysed using linear mixed-effect models that were implemented using the R packages *lme4*^62^ and *lmerTest*^63^. These variables included: 1) the observed (Sobs) numbers of bacterial and archaeal OTUs, 2) the predicted (Chao1) numbers of bacterial and archaeal OTUs, 3) bacterial and archaeal OTU-level diversity as represented by the Simpson’s Diversity Index, 4) total nematode abundance, 5) nematode family-level diversity as represented by the Simpson’s Diversity Index, 6) the abundances of nematode trophic groups and guilds, 7) FDA activity, and 8) beta-glucosidase activity. For these analyses, measurements of the response variable *y*_*itc*_ (for treatment *i* (*i*.*e*. herbicide), day *t* (days since treatment application), and container *c*) were assumed to follow the model:

$${y}_{itc}={\alpha }_{i}+{\beta }_{t}+{\gamma }_{it}+{a}_{c}+{\varepsilon }_{itc}$$

The terms *α*_*i*_, *β*_*t*_ and *γ*_*it*_ are fixed effects for treatment *i*, day *t* and their interaction, respectively. The random effect *a*_*c*_ accounts for the container of soil indexed by *c* from which repeated measurements were taken, which we assumed was independent (between containers) and normal with mean zero and variance *σ*_*c*_^2^. The residuals were also assumed independent and normal with variance *σ*^2^. The significance of treatment effects was assessed using F-tests.

Multivariate responses were analysed using permutational multivariate analysis of variance (PERMANOVA^64^) implemented using the R package *vegan*^65^. Multivariate analyses focussed on determining whether the treatments influenced: 1) the composition of bacterial and archaeal communities as represented by Hellinger transformed (square root of the relative abundances) OTU relative abundances^66^, 2) the composition of nematode communities as represented by the Hellinger transformed abundances of 14 nematode families, and 3) the substrate utilisation potential of microbial communities as represented by their induced respiratory responses to 15 substrates. All analyses were performed using R 3.2.3.
